# An Online Gravity Modeling Method Applied for High Precision Free-INS

**DOI:** 10.3390/s16101541

**Published:** 2016-09-23

**Authors:** Jing Wang, Gongliu Yang, Jing Li, Xiao Zhou

**Affiliations:** 1 School of Instrument Science and Opto-electronics Engineering, Beihang University, Beijing 100083, China; yanggongliu@buaa.edu.cn (G.Y.); buaa_lijing@buaa.edu.cn (J.L.); by1317110@buaa.edu.cn (X.Z.); 2 Inertial Technology Key Laboratory of National Defense Science and Technology, Beihang University, Beijing 100083, China

**Keywords:** polynomial model, high precision free-INS, online modeling, complexity analysis

## Abstract

For real-time solution of inertial navigation system (INS), the high-degree spherical harmonic gravity model (SHM) is not applicable because of its time and space complexity, in which traditional normal gravity model (NGM) has been the dominant technique for gravity compensation. In this paper, a two-dimensional second-order polynomial model is derived from SHM according to the approximate linear characteristic of regional disturbing potential. Firstly, deflections of vertical (DOVs) on dense grids are calculated with SHM in an external computer. And then, the polynomial coefficients are obtained using these DOVs. To achieve global navigation, the coefficients and applicable region of polynomial model are both updated synchronously in above computer. Compared with high-degree SHM, the polynomial model takes less storage and computational time at the expense of minor precision. Meanwhile, the model is more accurate than NGM. Finally, numerical test and INS experiment show that the proposed method outperforms traditional gravity models applied for high precision free-INS.

## 1. Introduction

Inertial navigation systems (INSs) are employed throughout all branches of the military and in many civil platforms, due to their significant advantages of providing continuous position, velocity and attitude information, and being invulnerable to external interference [1]. In the procedure of real-time navigation, the gravity acceleration is one of the significant pieces of external reference information needed for INSs [2]. This continuous gravity information is routinely obtained from the normal gravity model (NGM). With the development of inertial sensors, the availability of high-precision gravity information has become a crucial issue for preparing accurate INSs [3]. Therefore, a more accurate gravity description in high-precision INSs should be promoted. Besides online gravity models, en route gravity compensation methods using gradiometers are reported [4,5]. However, due to its independence from the accuracy of gravity sensitive instrumentation, gravity modeling has always been introduced into INSs [5,6].

As mentioned above, the normal gravity models (NGMs) described on the surface of reference ellipsoids are conventionally used to compensate for gravitational effects on accelerometers [7]. These models are usually expressed as simple combinations of first- and second-order sine functions. They are successfully applied for current low or medium accuracy INSs. However, for high-precision systems, the NGMs are inefficient. To increase NGMs’ accuracy, researchers have developed several improved NGMs [8,9,10], while the residual error of the normal gravity vector to the actual one (i.e., gravity disturbance vector, GDV) is still ignored. In the field of geodesy, spherical harmonic series, commonly used to represent the Earth’s gravitational field, are routinely expanded to ultra-high degree (>2160). In addition, the spherical harmonic gravity model (SHM) could describe the GDV accurately. That promotes the possibility of the application of spherical harmonic gravity model (SHM) for INSs to compensate for GDV-induced errors. However, we have demonstrated that only SHM of degree 12 or less is suitable for high frequency real-time solution of INSs, which still retains on average a 27.84 mGal horizontal gravity error globally [11]. Since SHM’s computational time increases quadratically with its degree, it greatly exceeds a single navigation period. According to our surveys, no studies have mentioned the application of high-degree SHM for INSs. Even so, the high-fidelity SHM has always attracted the attentions of scientists in astrodynamics to develop efficient models for rapid and precise orbit propagation.

To improve the efficiency of SHMs’ execution, 3D interpolation models perform more acceptably for the Earth’s application [12]. The modeling method, effectively a trade-off between speed and memory, stores nodal gravity field information on an equivalent spherical grid. This information is previously calculated with SHM on a computer. Then, a variety of interpolation methods are employed to solve the gravity for real-time orbit determination. Junkins first proposed this modeling approach in 1976. However, it was limited by the low quality of space-borne computers at the time, because it needs to store almost 30,000 coefficients [13]. In 1996, Hujsak introduced the concept of pseudocenter and achieved a 100-fold speedup but with consumption of precision compared to SHM of degree 70. The coordinates of the pseudocenter can be used to derive gravity information, which reduces the memory requirement to the order of several megabytes [14]. Recently, the developments of memory and processor technology applied for satellites have motivated more works on the 3D interpolation models. Interpolation methods, such as weighting functions, wavelets, B-splines and octrees, were employed in the models to reduce the computational complexity [12,15]. Efficient models, such as the cubed-sphere model and the fetch model, were established to balance accuracy with complexity. However, in principle these 3D models improve the calculation speed at the expense of storage, which is the most limited resource for navigation computers, contrary to space satellites. Therefore, for high-precision INS, the direct application of these efficient models will be limited.

With the motivation of compensating the GDV-induced error for accurate INSs, the high-fidelity SHM is an obvious choice. To apply the model for real-time navigation, especially for navigation in rugged regions, we will modify it as an online gravity model in this paper. Therefore, considering the advantages of 3D interpolation models, we propose a more time- and space-efficient online modeling method. The method combines a regional two-dimensional second-order polynomial model (PM) with an online strategy for the coefficients. The PM is selected to establish gravity on a 6′ × 6′ navigation area, where the components of GDV vary almost linearly with latitude and longitude. To achieve global navigation, the coefficients of the PM update as the vehicle gets close to the boundary of the area. The updating of coefficients and the switch of navigation area are executed in an external gravity computer, where the PM is fitted to nodal gravity information calculated by SHM. This design is superior to data-based compensation methods that occupy several times the memory resources (see [8]) of harmonic coefficients. Compared with 3D interpolation models, the proposed model possesses only twelve coefficients that require a few bytes of memory. Moreover, it performs more efficiently relative to the interpolation methods.

The outline of this paper is as follows: Section 1 is the Introduction; the definition of gravity disturbance vector and its expression as spherical harmonic series are reviewed briefly in Section 2; Section 3 gives a demonstration of variations of gravity components on a small-scale range, and a numerical test is carried out to verify the theoretical derivation. In Section 4, the strategy of the novel online modeling method and gravity compensation is designed. Experimental results and discussions of the proposed method applied for a real free-INS are shown in Section 5, and conclusions are presented in Section 6.

## 2. Gravity Model in High Precision INS

### 2.1. Definition of Gravity Disturbance Vector

**Figure 1:**
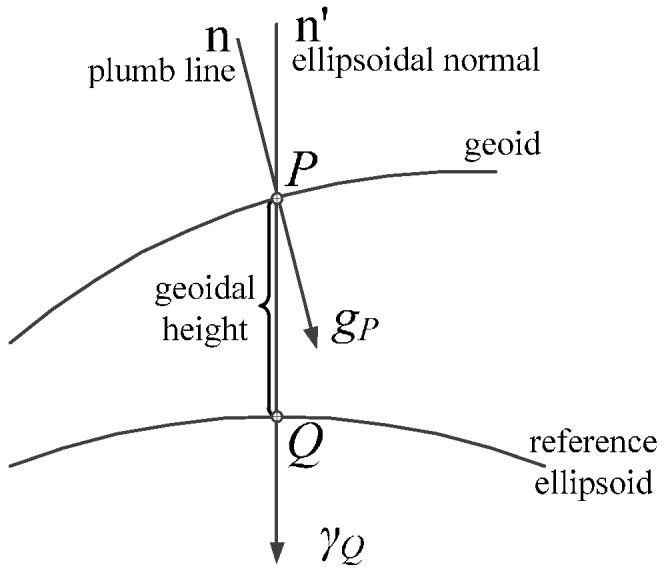
Description of gravity disturbance vector.

As Figure 1 shows, a point *P* on the geoid is projected onto the point *Q* on the surface of the ellipsoid by means of the ellipsoidal normal. The gravity disturbance vector (GDV) is defined as the difference between the gravity vector *g* at *P* and the normal gravity vector *γ* at the same point *P* [7]:

(1) $$\delta g=g_{P}-g_{P}$$

The difference in magnitude is the gravity disturbance, which historically presents as gravity anomaly Δ*g*, for it is more available and being processed than gravity disturbance:

(2) $$\Delta g=g_{P}-\gamma_{Q}$$

The difference in direction is the deflection of vertical (DOV), which has two components, a north-south component *ξ* and an east-west component *η*. Therefore, the GDV on the Earth’s surface for a high precision INS can be expressed as:

(3) $$\delta g^{n}=[\begin{array}{ccc} -\gamma \eta & -\gamma \xi & \Delta g \end{array}]$$

where, the superscript *n* denotes gravity defined in local geodetic frame. *γ* is the magnitude of the normal gravity vector *γ*, whose horizontal components on the ellipsoid’s surface are zero, i.e., ***γ*** = [0 0 –*γ*]. The GDV and normal gravity vector constitute the gravity vector compensated in high precision INS. The expressions of GDV’s three components obviously determine the accuracy and efficiency of the gravity model.

### 2.2. Spherical Harmonic Model

Commonly, the spherical harmonic expansion is used to represent the Earth’s gravitational field with high fidelity. The Earth Gravitational Model 2008 (EGM2008) computed by the U.S. National Geospatial-Intelligence Agency (NGA) is complete to degree 2160 [16]. It allows computing the disturbing potential and a number of gravity field related quantities, such as height anomalies, gravity anomalies and vertical deflections to a spatial resolution of about five arc minutes. Therefore, it is an optimal choice to invite the SHM for describing the GDV. Firstly, we write the expression of disturbing potential, of which the GDV is the gradient vector:

(4) $$T=-\frac{fM}{\rho }\sum_{n=2}^{\infty }{\left(\frac{a}{\rho }\right)}^{n}\sum_{m=0}^{n}\left({C^{\prime }}_{nm}\cos m\lambda +S_{nm}\sin m\lambda )P_{nm}(\cos\theta \right)$$

where, *f* is Newton’s gravitational constant; *M* is mass of the earth; *ρ* is the geocentric radius distance; *θ* is spherical polar angle; *λ* is the geocentric longitude; *C*′_nm_ and *S_nm_* are constant coefficients of the spherical harmonic of degree *n* and order *m*, among them *C*′*_nm_* is modified by reference ellipsoid parameters; *P_nm_* is the normalized associated Legendre function of the first kind. Then, we have the components of GDV on a computation point (situated at the Earth’s surface) as follows:

(5) $$\xi =\frac{1}{\gamma \rho }\frac{\partial T}{\partial \theta }=-\sum_{n=2}^{\infty }\sum_{m=0}^{n}({C^{\prime }}_{nm}\cos m\lambda +S_{nm}\sin m\lambda )\frac{dP_{nm}(\cos\theta )}{d\theta }$$

(6) $$\eta =-\frac{1}{\gamma \rho \sin\theta }\frac{\partial T}{\partial \lambda }=-\frac{1}{\sin\theta }\sum_{n=2}^{\infty }\sum_{m=0}^{n}m({C^{\prime }}_{nm}\sin m\lambda -S_{nm}\cos m\lambda )P_{nm}(\cos\theta )$$

(7) $$\Delta g=-\frac{\partial T}{\partial \rho }-\frac{2}{\rho }T=\gamma \sum_{n=2}^{\infty }\left(n-1\right)\sum_{m=0}^{n}\left(C\prime_{nm}\cos m\lambda +S_{nm}\sin m\lambda )P_{nm}(\cos\theta \right)$$

Accordingly, all components of the gravity model are expressed in the form of spherical harmonic series. This will impose a large computational complexity on the navigation solution. To retain the advantage of high-degree SHM and use it for a high precision INS, a simplified expression with less demands for accuracy should be investigated. Our following work will concentrate on these issues.

## 3. Mathematic Analysis of Spherical Harmonic Model

### 3.1. Gravity Disturbing Potential in Small Area

Since gravity disturbing potential formulation can be used to derive variations in gravity anomaly and vertical deflections, our discussions focus on Equation (4). When truncated at degree *n_max_*, the spatial resolution of the spherical harmonic expansion can be calculated as follows:

(8) $$\Delta \Psi =\frac{180{}^{\circ}}{n_{max}}$$

In the case of EGM2008, the maximum degree *n_max_* of 2160 implies a resolution of 5 arc minutes. Gravity disturbing potential of degree *n* is expressed as:

(9) $$T_{n}=\sum_{m=0}^{n}\left(a_{nm}\cos m\lambda +b_{nm}\sin m\lambda )P_{nm}(\cos\theta \right)$$

**Figure 2:**
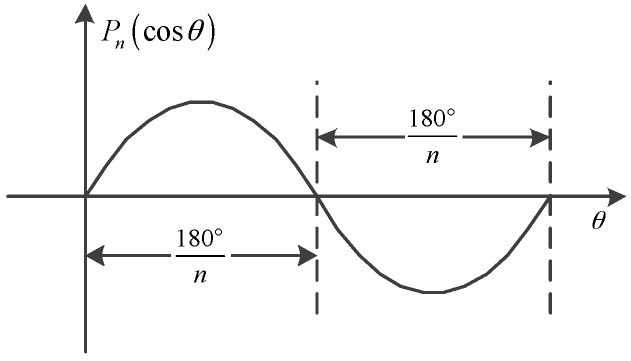
One period of function *P_n_*(cos*θ*).

The quantity of value of zero is at most *n* and 2*n* in the directions of *θ* and *λ*, respectively. The interval of these zeros in both directions is equal to 180°/*n*, i.e., the spatial resolution. That means the highest frequency of *T_n_* is *n*/2π. Figure 2 shows one period of function *P_n_*(cos*θ*) in the direction of *θ*.

In any 180°/*n* × 180°/*n* region of the sphere, the average gravity field is usually used for spherical harmonic synthesis instead of discrete values [17,18,19]. Therefore, we take the sense that some regular features would be explored from the gravity calculated with SHM in small size area. Here, we re-write the gravity disturbing potential as [20]:

(10) $$T=-\frac{fM}{\rho }\sum_{m=0}^{n_{\max}}c_{m}\cos m\lambda +s_{m}\sin m\lambda$$

with the lumped coefficients:

(11) $$\begin{array}{l} c_{m}=\sum_{n=\max\left(2,m\right)}^{n_{\max}}{\left(\frac{a}{\rho }\right)}^{n}{C^{\prime }}_{nm}P_{nm}\left(\cos\theta \right)\\ s_{m}=\sum_{n=\max\left(2,m\right)}^{n_{\max}}{\left(\frac{a}{\rho }\right)}^{n}S_{nm}P_{nm}\left(\cos\theta \right) \end{array}$$

In spherical harmonic analysis and synthesis, the surface of the Earth is gridded as numbers of subblock Δ*θ* × Δ*λ* [19]. We describe the disturbing potential values on a global grid as follows:

(12) $$T\left(k\Delta \theta ,l\Delta \lambda \right)=-\frac{fM}{\rho }\sum_{m=0}^{N}c_{m}\cos\left(ml\Delta \lambda \right)+s_{m}\sin\left(ml\Delta \lambda \right)$$

Then, we have its neighbor disturbing potential on the same latitude:

(13) $$T\left(k\Delta \theta ,\left(l+1\right)\Delta \lambda \right)=-\frac{fM}{\rho }\sum_{m=0}^{N}c_{m}\cos\left(m\left(l+1\right)\Delta \lambda \right)+s_{m}\sin\left(m\left(l+1\right)\Delta \lambda \right)$$

Considering the variation between the two points, we make a subtraction:

(14) $$T\left(k\Delta \theta ,\left(l+1\right)\Delta \lambda \right)-T\left(k\Delta \theta ,l\Delta \lambda \right)=-\frac{fM}{\rho }\sum_{m=0}^{N}2\left(c_{m}\cos\left(m\left(l+\frac{1}{2}\right)\Delta \lambda \right)+s_{m}\sin\left(m\left(l+\frac{1}{2}\right)\Delta \lambda \right)\right)\sin\frac{m\Delta \lambda }{2}$$

**Figure 3:**
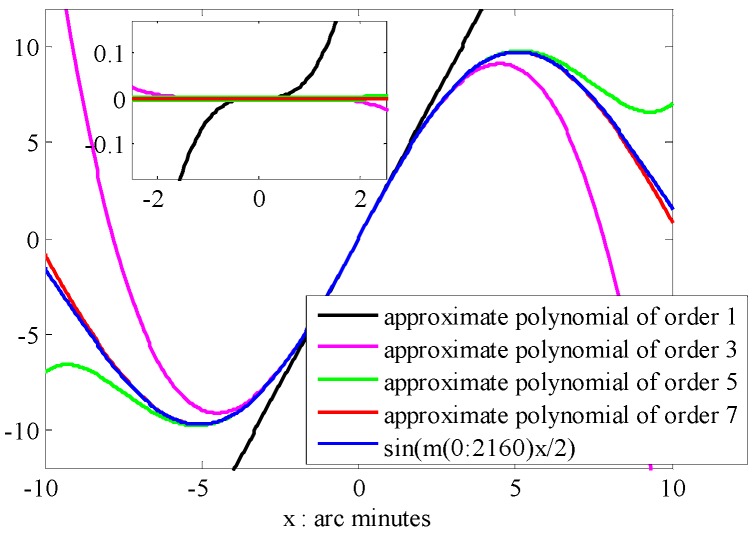
Approximation of sin(*m*Δ*λ*/2) as Δ*λ* = −5′~5′ and *m* = 0~2160.

This difference oscillates at the frequency *m*/4π, which will vary with the quantity of *m*. The highest frequency is at the point *m* = 2160, in the case of EGM2008. Thus, the level of nonlinearity for this difference is determined by the sum of sin(*m*Δ*λ*/2), where *m* ∈ [0, 2160]. Then, we turn our focus to the grid interval Δ*λ*. If it is less than the model’s resolution, i.e., Δ*λ* < Δ*Ψ*, the value of *m*Δ*λ*/2 will be less than π/2 at *m* = 2160. That means the sine function will be a small value that could be simply treated as a linear or low order polynomial, when Δ*λ* = Δ*Ψ*/2. For the sum of sin(*m*Δ*λ*/2), all of the calculated angles are less than π/4. Assuming the coefficients of sine functions to be a series of normalized random data in the range of [−1, 1], the sum of sin(*m*Δ*λ*/2) is shown in Figure 3 coupled with its fitted polynomial functions of different order. The inset of Figure 3 shows the differences between the regressed functions and the basic function.

It is observed that the residual error of a third-order polynomial function fitted to the sum of sine functions is nearly 0.02. This is less than 0.2 percent of the peak value. The polynomial functions of order greater than three performed better when the interval Δ*λ* is in the range of [−2.5, 2.5] arc minutes. Similarly, we could obtain the same results on the direction of Δ*θ* according to the property of normalized associated Legendre function *P_nm_*(cos*θ*). Therefore, we propose that two-dimensional polynomials (for the model is established on the Earth’s surface, we consider the coordinate in the vertical direction to be constant here) of low order could be used to fit to any components of GDV in the area with size of half of the SHM’s resolution. Numerical tests address this in the following section.

### 3.2. Numerical Tests

#### 3.2.1. Test Design

To illustrate the theory above, two test areas are chosen, one in the North China Plain and another in the Himalayas. Based on EGM2008 and maximum degree 2160, 900 and 360, basic values of DOVs are generated on dense grids with 50 × 50 points. In addition, the database is obtained on three subregions of each test area, sizes of which are corresponding to 15′ × 15′, 6′ × 6′ and 2.5′ × 2.5′. A remark made here that we discuss the horizontal components of GDV only, because the vertical channel of INS normally requires some external aiding, such as barometric altitude [21].

The 50 × 50 DOVs of each area are shown in Table 1 and Table 2. Recollecting DOVs on 15 × 15 knots of above areas, we fit two-dimensional second- and third-order polynomials to them. And then, DOVs on each 50 × 50 grids generated from PMs could be used to compare with the above database.

#### 3.2.2. Test Results

Subtracting DOVs calculated by the PMs from database, we get the root mean square (RMS), the maximum and minimum values of the approximation errors. Table 3 reports the descriptive statistics for the North China Plain and Table 4 for the Himalaya region.

In both test areas, the approximation errors show the same level of accuracy for subregions whose sizes are equal to half of the SHM’s resolution. Specifically, using a second-order polynomial produces the RMS-errors of 10^−2^ arc·s in smooth area and 10^−1^ arc·s in rugged area. A third-order polynomial reduces the approximation errors to 10^−3^ and 10^−2^ arc·s, respectively. As the size is reduced, the approximation accuracy improves.

Actually, a 0.1 arc·s DOV induces a position error on the order of several meters per hour [22]. Therefore, to involve a larger applicative area of the fitted polynomial, we set this 0.1 arc·s DOV as a permission index of model approximation. Further, this index could theoretically satisfy the accuracy demand of a modern high-precision navigation system.

## 4. Online Gravity Modeling in INS

**Figure 4:**
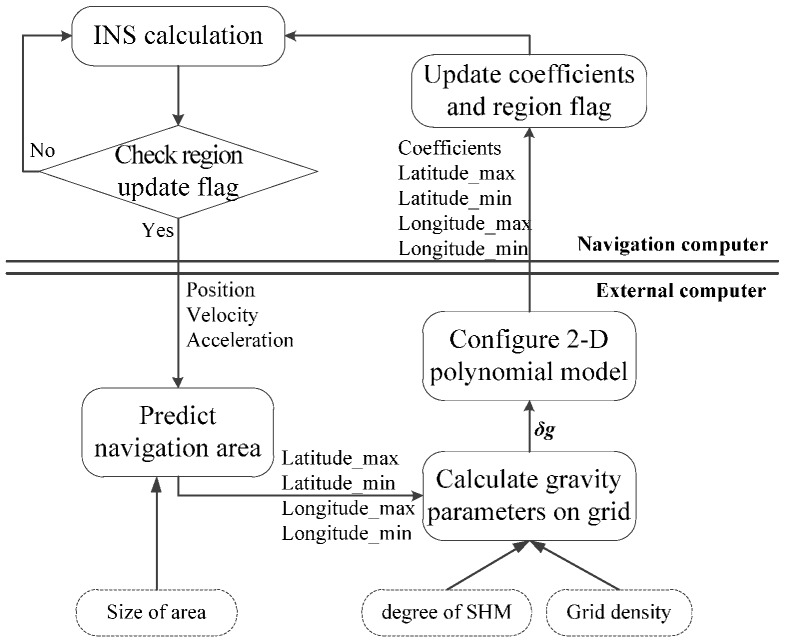
The scheme of model configuration in INS.

Since the two-dimensional polynomial of low order represents the regional gravity precisely, we propose the online gravity modeling scheme shown in Figure 4.

As it shown, our navigation system comprises the navigation computer and the gravity computer [2,23]. The online modeling method provides PM for dynamic INS in real-time and global navigation. To update PM for varying navigation areas, the method will be aided by the external gravity computer. Once the vehicle is close to the boundary of the current area, the coefficients of PM and next applicable area will be updated in the external computer. The modeling scheme is implemented as follows:

*Step 1*: INS gives the motion information of vehicle to the external computer, consisting of position, velocity and acceleration.
*Step 2*: The prediction module calculates the applicative area of the updated model, concluding maximum latitude, longitude and minimum latitude, longitude.
*Step 3*: Program of SHM computes DOVs on grid points in batches.
*Step 4*: The configuration of the 2-D PM gives coefficients of updated models by fitting low order PM to the DOVs in step 3. This parameters combined with the position range of applicative area are transferred to the INS. The applicative area updates once the vehicle travels close to the boundary.

The prime parameters for model configuration in the external computer are the degree of SHM, size of area and grid density, which we will discuss in the following section.

### 4.1. Model Configuration

#### 4.1.1. Degree Selection of Base Model

The SHM as the base model has two types of errors: commission errors and omission errors. The commission errors are due to incorrectly estimating coefficients of the model, and the omission errors are due to truncation errors of the short wavelength variations of the gravity potential which are not modeled [15,24]. The degree variances of DOVs and error degree variances are used to describe these errors, whose expressions are as follows:

(15) $$\nu^{2}=M\left\{\xi^{2}+\eta^{2}\right\}=\sum_{n=2}^{\infty }n\left(n+1\right)\sum_{m=2}^{n}\left({C\prime }_{nm}^{2}+S_{nm}^{2}\right)$$

(16) $$\delta \nu^{2}=\sum_{n=2}^{\infty }n\left(n+1\right)\sum_{m=2}^{n}\left(\delta {C\prime }_{nm}^{2}+\delta S_{nm}^{2}\right)$$

**Figure 5:**
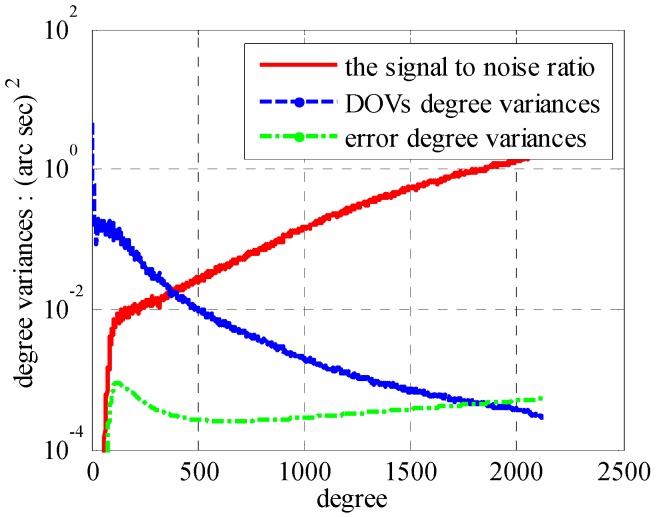
The DOVs degree variances.

In the case of EGM2008, the DOVs degree variances, error degree variances and their ratio are plotted in Figure 5.

**Figure 6:**
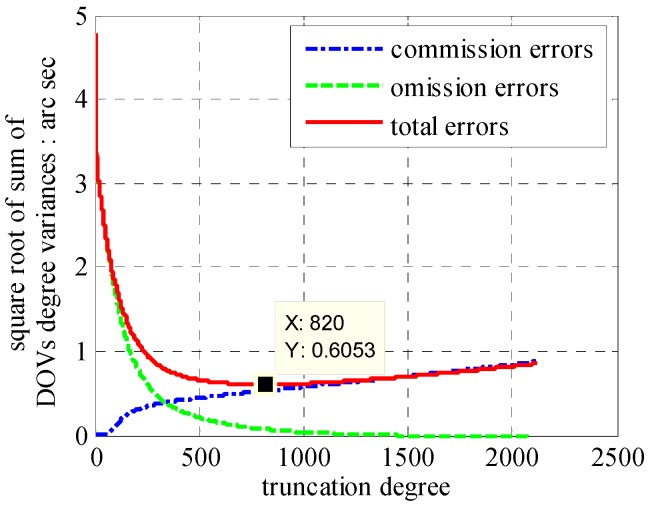
Errors of SHM with degree truncated to 3:2160.

According to Figure 5, the signal to noise ratio (SNR) becomes 1 near degree 1840 and 0.1 near degree 900. Compared with SHM of degree 2160, the commission errors and omission errors in Figure 6 illustrate the smallest value in degree 820. Combining with our previous research that only one-third omission errors occupied by degree from 141 to 2160 [10], we choose SHM of degree 820 as our base model here. This model has a SNR of 0.08, and will guarantee the computational efficiency at the expense of a little accuracy. In this case, the commission error is 0.53 arc·s. In addition with the omission error of 0.08 arc·s, it yields a total error of 0.61 arc·s. Therefore, added to the polynomial approximation error of 0.1 arc·s, the 0.71 arc·s error will induce position error ranging from ten meters to thirty meters [22]. For an INS with precision demand 0.2 nm/h, the PM-induced residual error accounts for less than ten percent of the total position error.

#### 4.1.2. Configuration of Polynomial Model

As mentioned in the preceding section, the main focus of our modeling method is to reconfigure SHM of degree 820 with a low order polynomial for local areas. The resolution of the model is about 13.2 arc·min. According to the analysis of Section 3, the size of the local area should not more than 6.6 arc·min. To facilitate the configuration, we round this value down to 6 arc·min.

Subdividing the 6′ × 6′ area into several subintervals with equal angle along latitude and longitude, we obtain DOVs on the grid calculated by SHM. These basic values are utilized to configure our polynomial model.

**Figure 7:**
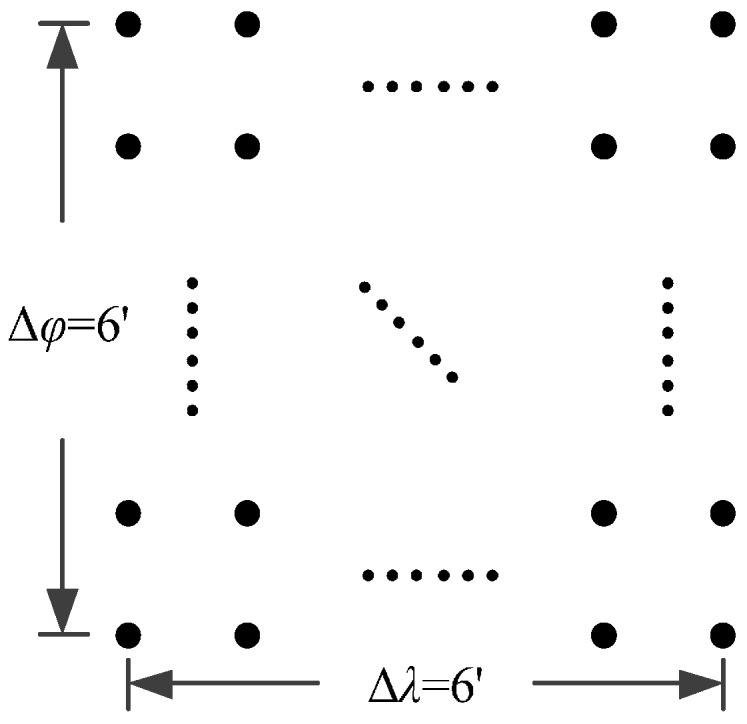
The subdivision of 6′ × 6′.

**Figure 8:**
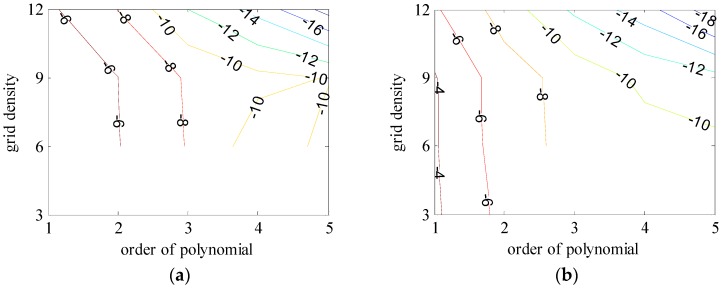

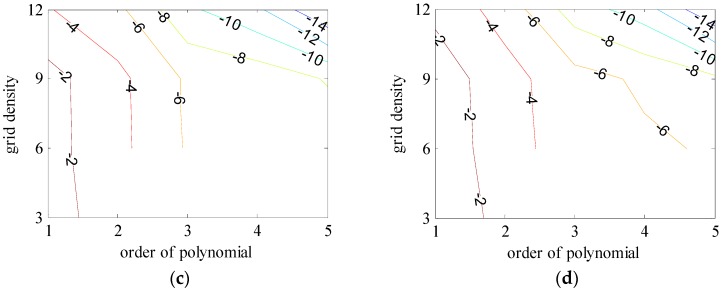
The fitted error of PMs of order 1 to 5 in two test areas with knot density of 3, 6, 9 and 12. (**a**) lg(RMS) of xi in North China Plain; (**b**) lg(RMS) of eta in North China Plain; (**c**) lg(RMS) of xi in the Himalayas; (**d**) lg(RMS) of eta in the Himalayas.

On the other hand, except for accuracy, the execution time of model configuration dominates our designation. A proper order of polynomial and knot density of area should be analyzed in modeling procedure. They are the primary limitations of the execution time for model preparation. To investigate these values, we establish PM on the subdivision as Figure 7 shows:

The basic data chosen here is DOVs from two 6′ × 6′ areas in Section 3.2, which are calculated by the base model. We fit first- to fifth-order PMs to the grid DOVs, whose number is *k* × *k*. In the test, the value of *k* is set as 3, 6, 9 and 12 respectively. The performances of PMs are tested through results of ten million points in each test area. The descriptive statistics (lg(RMS)) of the polynomial approximate results minus basic data are reported in Figure 8.

In Figure 8, the contour label-2 denotes the RMS of errors approximated with PM is 0.01 arc·s. According to the result of fitting a second-order polynomial to the 3 × 3 grid DOVs, the RMS error could reduce to less than 0.1 arc·s in both the smooth area and rugged area. Therefore, when we choose the order of polynomial as 2 and the grid density as 3, the accuracy requirement of INS could be fulfilled greatly.

Finally, our approximation formulae of DOVs expressed as second-order polynomial of two dimensions are as follows:

*ξ* assumes the form:

(17) $$\xi =\sum_{i=0}^{2}\sum_{j=0}^{2-i}a_{ij}\lambda^{i}\varphi^{j}$$

*η* assumes the form:

(18) $$\eta =\sum_{i=0}^{2}\sum_{j=0}^{2-i}b_{ij}\lambda^{i}\varphi^{j}$$

**Figure 9:**
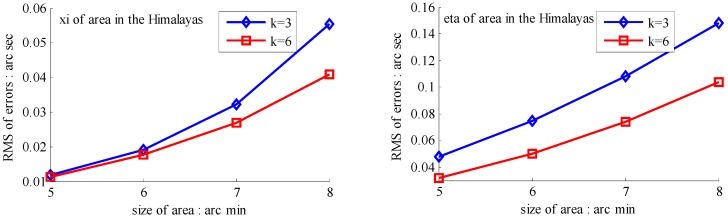

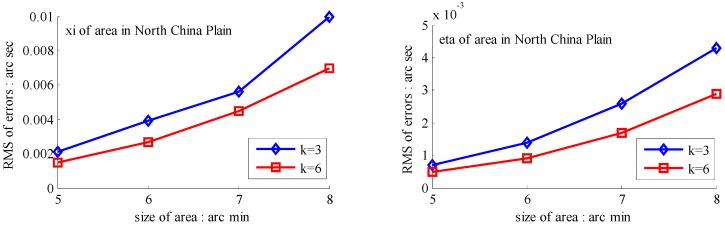
The RMS of fitted errors for different size of area, when k = 3 and 6.

The coefficients *a_ij_*, and *b_ij_* (*i*, *j* = 0,1,2) will be determined by disturbance components at 3 × 3 points of predicted 6′ × 6′ navigation area. An additional topic that has to be presented here is the balance between size of area and grid density. During the update of model coefficients in the external computer, we hope it takes as little computational time as possible. Accordingly, we should choose a small value of *k*, based on which the size of area should be as large as possible. Taking the two test areas for example, we calculated the RMS results of errors for different size of area, when *k* = 3 and 6. As Figure 9 shows, a 6′ × 6′ update area of model is the best choice for the approximate accuracy of 0.1 arc·s.

### 4.2. Computational Complexity

The computational complexity of a model itself should be of concern except for its precision. In this section, we analyze the time and space complexity of the proposed modeling method. The arithmetic operations involved include addition (ADD), multiplication (MUL), division (DIV) and comparison (COMP). Considering the procedure of model configuration mentioned above, our online modeling method concludes two parts: one is the real-time computation of gravity vector for free-INS, and the other is the synchronous update of PM’s coefficients in external computer.

It is obvious that there is two second-order polynomials and twelve coefficients in the real-time model computation. Therefore, the total operations of real-time model computation are 10ADD+18MUL, and the space complexity is 12 float. Storage taken by the model in navigation computer is approximate 0.09 KB. Tests for timing and accuracy reported in Table 5 were done by comparing performance of PM to routine NGM, modified NGM proposed in reference [8], the Cubed-Sphere model and SHM of degree 12 and 820. The test consists of computing gravity vectors for 10,000 randomly generated points, then comparing average execution times and accuracy. The speed-up factor in the fifth column is obtained by dividing the execution time for the SHM of degree 820 by the execution times for other models. The l2-norm of the difference between the gravity vectors produced by the first five models and those of SHM of degree 820 are reported, whose largest errors are in the sixth column. Observe that, the proposed PM performs outstandingly in both accuracy and execution time.

For the operation of updating the model’s coefficients, computations of nine points with SHM of degree 820 are needed. Because the *c_m_*, *s_m_* are independent of *λ*, Equation (11) needs to be evaluated only once for computation points densely spaced along a parallel (i.e., *φ* constant), and the efficiency can be further increased for points equally spaced in longitude [17]. According to the time complexity of a single point analyzed in our previous work, the operations of the 3 × 3 grids are approximate to (13ADD+18MUL+3DIV+1COMP)(*n_max_* + 2)(*n_max_* − 1), where *n_max_* is equal to 820. Compared with this process of gravity computation, the execution time of the fit algorithm to discrete data could be ignored. Without additional stored parameters, the space complexity of execution of coefficients update is equal to 2(*n_max_* + 4)(*n_max_* − 1) float. When *n_max_* = 820, the storage occupied is 11.4 MB. Toward a processor with 2.6 GHz master frequency, the computation time is on average approximately 2.54 s. It means that the prediction of navigation area should be finished to retain the update time for model coefficients before the vehicle reaches the boundary of the present navigation area.

## 5. Experimental Results

**Figure 10:**
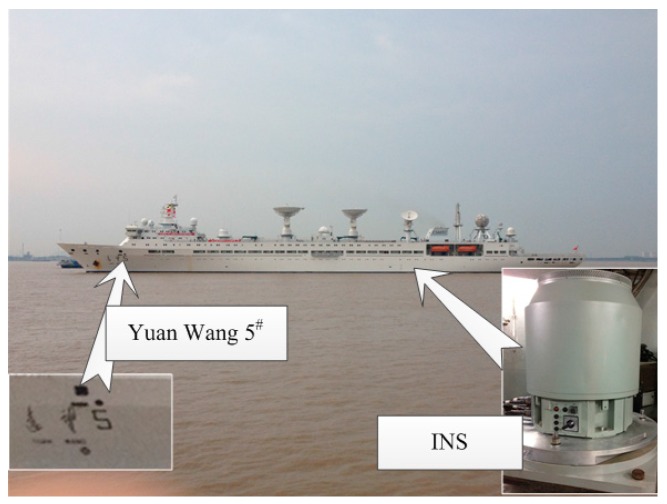
Ship test in the South China Sea.

**Figure 11:**
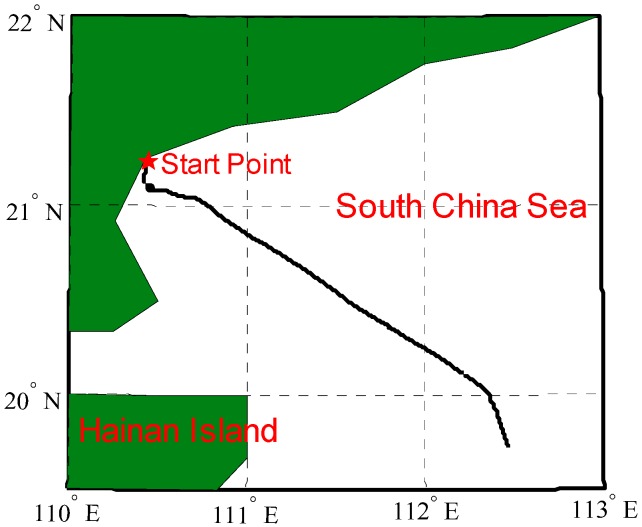
Trajectory of the ship test.

**Figure 12:**
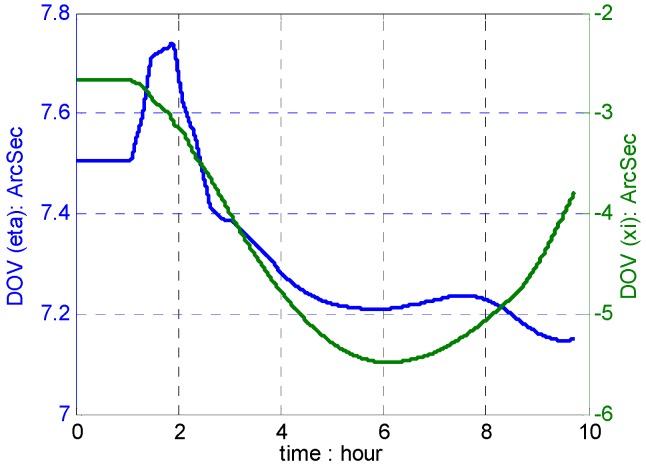
DOVs on the trajectory.

To illustrate the outstanding performance of PM in free-INS, a ship test was performed in the South China Sea using the Yuan Wang 5# surveying ship (shown in Figure 10). The high-precision strapdown INS used in the test consists of three ring laser gyroscopes with bias stability of 0.003 °/h and three quartz accelerometers with bias stability of 5 µg, where PM is carried out. The travel profile and DOVs on the trajectory are shown in Figure 11 and Figure 12.

**Figure 13:**
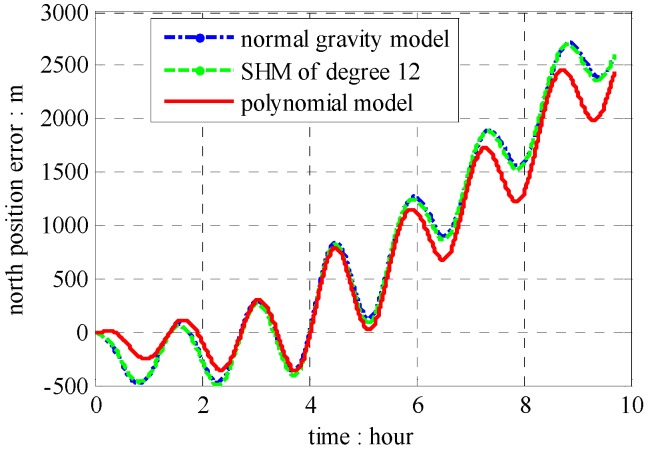
Position errors of north components.

**Figure 14:**
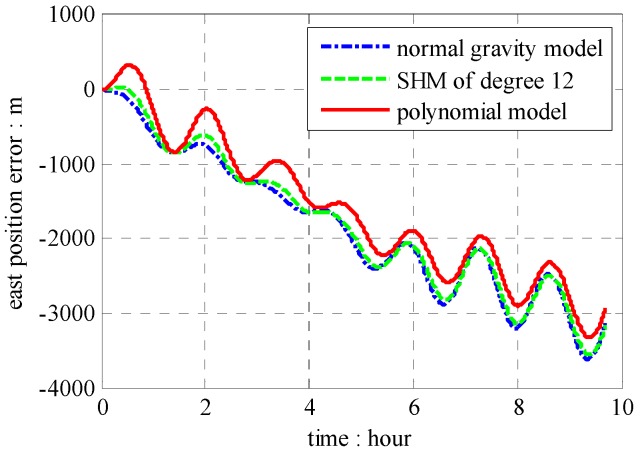
Position errors of east components.

**Figure 15:**
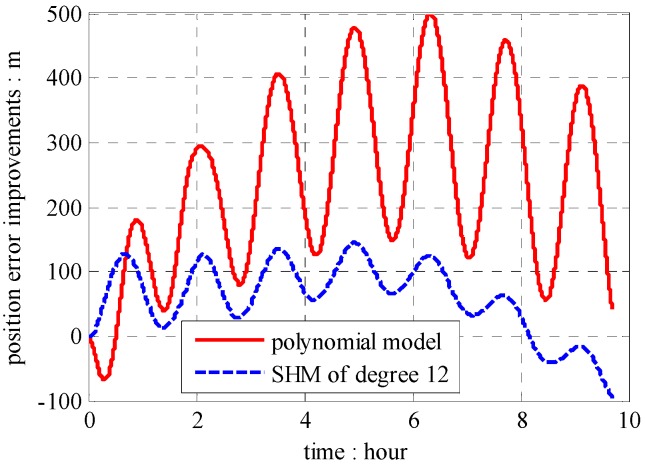
Square root of position error components.

The position results compared with GPS data are shown in Figure 13, Figure 14 and Figure 15, which combined with results induced by traditional NGM and SHM of degree 12.

According to results in Figure 13, Figure 14 and Figure 15, the proposed polynomial model has the best performance compared with the NGM and SHM of degree 12. Relative to NGM, the maximum value of position error improvements is 500 m during the ten hours travel when navigation scheme is compensated by the polynomial model, while, it is 152 m in the navigation scheme compensated by the SHM of degree 12. Therefore, the effectiveness of the online gravity polynomial model is verified in that it could improve the position error of ship-borne INS by about ten percent.

## 6. Conclusions

The main motivation of this paper is to provide an efficient method for the application of high-degree SHM in free-INS. Due to time and space limitations, the high-degree SHM is not suitable for real-time navigation solutions. To reduce the computational complexity, we firstly propose an approximation model that converts the global support SHM to local support. Mathematical analysis illustrates the high accuracy of a low order polynomial approximating the SHM in a small area. Numerical tests in two typical areas (the North China Plain and the Himalayas) show that the DOVs’ approximation errors of a second-order polynomial are as much as 0.01 arc·s in a smooth area and 0.1 arc·s in a rugged area than full degree SHM. To accommodate the real-time navigation, we propose a novel navigation scheme assisted with an external gravity computer in which the coefficients of the polynomial model will update synchronously. Further studies demonstrate that the optimal degree of basic SHM and the number of grid points for fitted coefficients are 820 and 3 × 3, respectively. Combined with the theoretical analysis, the proposed gravity model is finally expressed as a second-order polynomial in each 6′ × 6′ navigation area. Computational complexity and largest l2-norm error of the model are very low as compared with the traditional NGM, typical modified NGM, the cubed-sphere model, and SHM of degree 12 and 820. The efficiency of polynomial model is verified in a ship test. Compared with traditional NGM, the maximum position errors in the travel profile were decreased by ten percent.

## Figures and Tables

**Table 1:** DOVs of different area sizes in the North China Plain.

Size of Area	DOVs	2160	900	360
15′ × 15′	*ξ*	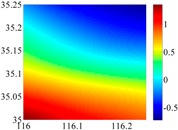	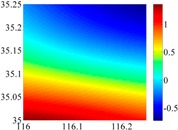	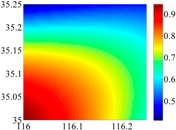
	*η*	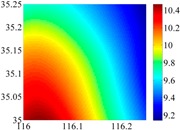	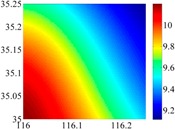	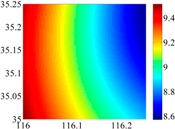
6′ × 6′	*ξ*	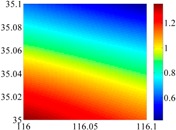	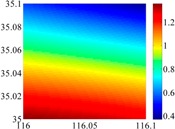	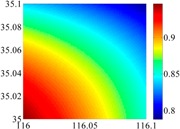
	*η*	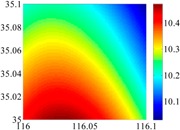	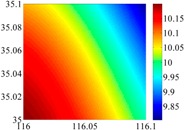	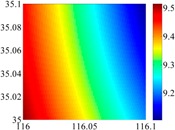
2.5′ × 2.5′	*ξ*	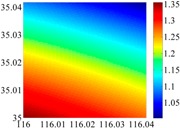	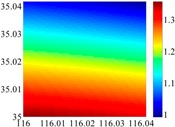	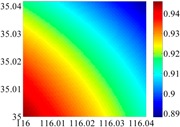
	*η*	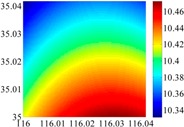	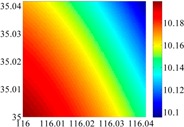	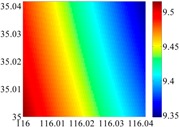

**Table 2:** DOVs of different area sizes in the Himalayas.

Size of Area	DOVs	2160	900	360
15′×15′	*ξ*	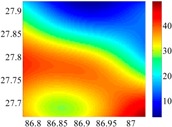	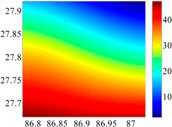	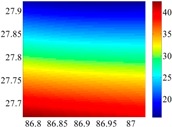
	*η*	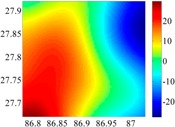	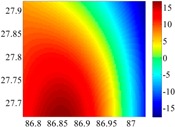	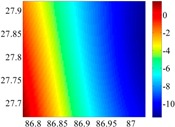
6′×6′	*ξ*	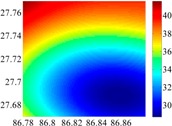	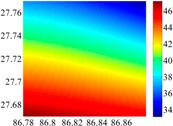	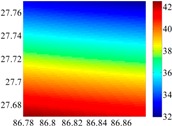
	*η*	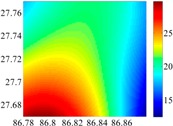	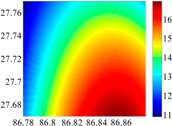	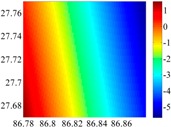
2.5′×2.5′	*ξ*	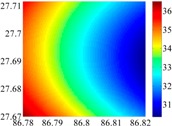	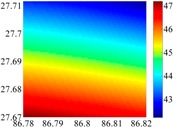	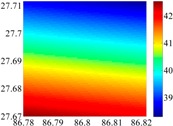
	*η*	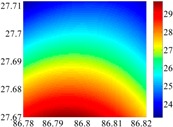	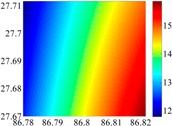	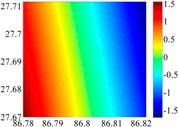

**Table 3:** Numerical results of polynomial fitting in the North China Plain.

Size of Area	Degree of Polynomials	DOVs	2160			900			360		
			RMS	Max	Min	RMS	Max	Min	RMS	Max	Min
15′ × 15′	2	*ξ*	0.0270	0.0534	−1.259	0.0231	0.0516	−0.1051	0.0035	0.0121	−0.0130
		*η*	0.0263	0.0864	−0.1458	0.0192	0.0750	−0.1058	0.0043	0.0128	−0.0234
	3	*ξ*	0.0179	0.0406	−0.0660	0.0087	0.0140	−0.0300	6.84 × 10^−4^	0.0056	−0.0013
		*η*	0.0106	0.0387	−0.0385	0.0032	0.0074	−0.0190	8.64 × 10^−4^	0.0016	−0.0067
6′ × 6′	2	*ξ*	0.0043	0.0159	−0.0182	0.0036	0.0123	−0.0134	3.06 × 10^−4^	8.75 × 10^−4^	−8.60 × 10^−4^
		*η*	0.0050	0.0190	−0.0269	0.0018	0.0080	−0.0073	2.98 × 10^−4^	0.0011	−0.0014
	3	*ξ*	0.0014	0.0054	−0.0060	1.39 × 10^−4^	4.55 × 10^−4^	−6.68 × 10^−4^	1.49 × 10^−5^	1.16 × 10^−4^	−2.71 × 10^−5^
		*η*	7.64 × 10^−4^	0.0017	−0.0045	7.26 × 10^−5^	5.18 × 10^−4^	−2.92 × 10^−4^	2.0 × 10^−5^	3.67 × 10^−5^	−1.44 × 10^−4^
2.5′ × 2.5′	2	*ξ*	4.10 × 10^−4^	0.0022	−0.0023	2.67 × 10^−4^	0.0011	−0.0011	2.37 × 10^−5^	6.84 × 10^−5^	−6.82 × 10^−5^
		*η*	4.37 × 10^−4^	0.0024	−0.0022	1.19 × 10^−4^	5.79 × 10^−4^	−5.87 × 10^−4^	2.16 × 10^−5^	8.72 × 10^−5^	−9.52 × 10^−5^
	3	*ξ*	4.67 × 10^−5^	2.47 × 10^−4^	−9.65 × 10^−5^	4.66 × 10^−6^	3.09 × 10^−5^	−1.08 × 10^−5^	4.04 × 10^−7^	3.13 × 10^−6^	−7.0 × 10^−7^
		*η*	2.50 × 10^−5^	9.93 × 10^−5^	−6.13 × 10^−5^	3.44 × 10^−6^	2.63 × 10^−5^	−6.81 × 10^−6^	5.64 × 10^−7^	8.95 × 10^−7^	−4.14 × 10^−6^

**Table 4:** Numerical results of polynomial fitting in the Himalayas.

Size of Area	Degree of Polynomials	DOVs	2160			900			360		
			RMS	Max	Min	RMS	Max	Min	RMS	Max	Min
15′ × 15′	2	*ξ*	3.8582	16.1806	−12.2163	0.4304	2.6393	−1.8167	0.0718	0.2832	−0.3048
		*η*	3.3621	9.4731	−9.1792	1.0048	4.9811	−2.4415	0.0666	0.3633	−0.3771
	3	*ξ*	2.6084	15.6082	−5.6079	0.1389	1.0797	−0.4653	0.0030	0.0113	−0.0133
		*η*	1.9458	7.4299	−5.2106	0.2373	1.5610	−0.4497	0.0046	0.0070	−0.0346
6′ × 6′	2	*ξ*	0.5512	1.7377	−2.6511	0.0405	0.1836	−0.1771	0.0044	0.0202	−0.0200
		*η*	0.2777	1.3142	−1.0185	0.0823	0.4472	−0.4400	0.0048	0.0259	−0.0260
	3	*ξ*	0.1143	0.1969	−0.9933	0.0024	0.0171	−0.0071	9.82 × 10^−5^	3.99 × 10^−4^	−3.1 × 10^−4^
		*η*	0.1453	0.3523	−1.0071	0.0050	0.0385	−0.0083	1.02 × 10^−4^	2.13 × 10^−4^	−7.57 × 10^−5^
2.5′ × 2.5′	2	*ξ*	0.0334	0.1172	−0.1594	0.0033	0.0139	−0.0135	3.08 × 10^−4^	0.0014	−0.0014
		*η*	0.0504	0.2311	−0.2644	0.0069	0.0350	−0.0354	3.59 × 10^−4^	0.0019	−0.0019
	3	*ξ*	0.0035	0.0064	−0.0265	5.16 × 10^−5^	2.49 × 10^−4^	−1.82 × 10^−4^	3.23 × 10^−6^	1.31 × 10^−5^	−9.38 × 10^−6^
		*η*	0.0027	0.0093	−0.0202	9.39 × 10^−5^	5.6 × 10^−4^	−2.98 × 10^−4^	2.71 × 10^−6^	6.41 × 10^−6^	−1.9 × 10^−5^

**Table 5:** Comparison of models’ performances.

Model	Time Complexity	Space Complexity	Execution Time (s)	Speed-up Factor	Largest l2 Error (m/s^2^)	Real-Time Navigation
PM	10ADD+18MUL	12 float	3.9868 × 10^−6^	7.5381 × 10^5^	3.2368 × 10^−6^	Available
NGM	9ADD+11MUL	3 float	2.9605 × 10^−6^	1.0151 × 10^5^	0.0130	Available
Modified NGM	9ADD+10MUL	3 float	2.2697 × 10^−6^	1.3243 × 10^6^	0.0130	Available
12 degree SHM	O(12^2^)	352 float	0.0028	1.0733 × 10^3^	1.9897 × 10^−4^	Available
Cubed-sphere model (*m* = 11 and *N* = 2048)	O(12^3^)	1,226,492,416 float	0.0181	1.6604 × 10^2^	2.3785 × 10^−6^	Unavailable
820 degree SHM	O(820^2^)	572,137,984 float	3.0053	1	0	Unavailable

*m* is degree of B-spline, and *N* is resolution of the cubed sphere.
